# Hospital and Institutional News

**Published:** 1915-04-10

**Authors:** 


### HOSPITAL AND INSTITUTIONAL NEWS.

### "THE HOSPITAL'S" SPECIAL NUMBER ON "TRANSPORT."

We hope to publish next week a Special Number of The Hospital devoted to the subject of Transport, which, it is not too much to say, has been the central problem of the campaign as much in its military as in its medical aspects. Confining ourselves to the transport of wounded, our endeavour to secure first-hand information from those actively engaged in the superintendence of the work at all the main points in the long chain of transit which conducts the wounded from the trenches to hospitals in England met with many difficulties which will be obvious to our readers. The result, however, has been what we desired, and among the chief articles of interest are the following: Sir George Beatson, K.C.B., chairman of the Scottish Branch of the Red Cross Society, contributes a long article on "The Feeding and Comforts of the Wounded during Transit," in which the central principles of transport are discussed in the light of the present campaign. An insight is given into the plan on which are based those operations, of which we receive isolated glimpses at ports and railway stations. Then the practical aspects of the work of transport in its several links are considered in articles written by those actively engaged in the work. "Life and Procedure on a Hospital Ship" are described vividly and carefully by a lieutenant in the Royal Army Medical Corps, for permission to make use of whose special contribution we are much indebted to the officer commanding the ambulance ship on board which the writer has been working since the outbreak of war. Another article deals with "The Legal Status of the Hospital Ship," which is a subject of more than academic interest now that the force of international law is so much in question. "The Organisation of a Red Cross Train," with illustrations, provides another remarkable description of a section of the transport work, and to the last link in the transport service, the work of the Voluntary Aid Detachments, a special article, also illustrated, is devoted, by Mr. A. W. Faire, County Director of the Voluntary Aid Detachments, Leicester. The ship, the train, the ambulance and stretcher work of the Voluntary Aid Detachments, with some account of the rest stations, are thus authoritatively dealt with; while an account is also given of a Red Cross Field Kitchen at the Front, which plays so important a part in the work. A first-hand view is thus given of the whole complex organisation by which the wounded are brought to our hospital doors, the successful working of which has been one of the triumphs of the war. The price of the paper, notwithstanding its special features, will be, as usual, one penny.

### THE OVERWORKING OF INFIRMARY MEDICAL OFFICERS.

The difficulty in obtaining medical officers and one method of meeting it is illustrated by the experience of the Stoke Board of Guardians. Advertisements for an assistant medical officer at £160 per annum, and afterwards at £200, failed to elicit any response. Meanwhile the work at the infirmary was carried on single-handed by the chief medical officer, who was finally compelled to report to the Guardians that he found the task impossible. In the course of his report he said, "There are at present 447 patients in the hospital, many of them acutely ill, and 541 patients in the remainder of the house, the majority of whom are old and infirm and constantly require more or less medical supervision. To administer the institution properly, to dispense the medicines, and to perform the clerical work now required is a sheer impossibility for any individual." At their last meeting the Guardians decided to appoint a local practitioner to attend for two hours on three days weekly at a fee of two guineas per week. It is obvious that even with this assistance the work cannot be carried out properly. Further assistance should be given by the appointment of a dispenser and a clerk.

### POOR-LAW MEDICAL OFFICERS AND CLERICAL WORK.

The resignation of Dr. Robson, the medical officer of Alnwick Workhouse, owing to the amount of clerical work required by the Poor-Law Institutions Order, has been averted by the Guardians adopting the suggestion we made in our issue of January 30. Dr. Robson's salary has been increased from £80 to £90 a year, and the Guardians have requested the workhouse committee to consider the appointment of a clerk for the medical officer and workhouse master. At Lincoln, where a somewhat similar position has arisen, the Guardians have attempted to solve the difficulty by means which the Local Government Board has rightly refused to sanction. The Guardians suggested that the superintendent nurse should fill up the record papers. This is obviously wrong. The clinical notes must be kept by a medical man, and the increased work in this respect must be arranged for either by raising the salary of the medical officer, so as to enable him to devote more time to the work, or by appointing an assistant medical officer.

### WOMEN AS SANATORIA MEDICAL OFFICERS.

The one branch of medicine which has as yet not opened its doors much more widely to women on account of the scarcity of medical men has been tuberculosis; but signs are not wanting that it, too, will soon be offering increased opportunities to women doctors. Only a few weeks ago the Staffordshire, Wolverhampton, and Dudley Joint Committee for Tuberculosis discussed the advisability of appointing a woman doctor to the post of resident medical officer at Groundslow House Sanatorium.

It is interesting to note that the proposal was put forward by a medical man, who was, of course, able to quote several instances of good work being performed by women house surgeons at Wolverhampton institutions. The speaker even added that a woman resident doctor at the sanatorium would be less expensive, but that is perhaps open to doubt in view of the difficulty of obtaining anyone. The real difficulty of the appointment of women to resident medical posts in sanatoria is that no merely medical qualifications are sufficient, since the adequate control and administration of tuberculosis patients—many of whom are, of course, not confined to wards—is a problem in itself requiring special experience, if not special personal qualities, for its successful management. If anyone still doubts this he has only to recall one or two cases of difficulty and "revolt" which occurred soon after the appointment of practitioners, with no special experience in this regard, to the resident posts in those sanatoria which were opened after the passing of the Insurance Act. That women can be very successful administrators no one doubts, and if we make no beginning one cannot tell how successful they may be in this direction also. But the problem presented by opening sanatoria resident medical officers' posts to women is a real one, and care should be exercised both as to the type of sanatorium and to the personality of the woman doctor in any case where the appointment of a woman is decided on.

### ST. THOMAS'S HOSPITAL HOSTEL.

The amenities of Lambeth Palace Road are said to be threatened by the hostel which St. Thomas's Hospital proposed to build on an island site in that thoroughfare, and the governors have been asked to make other arrangements so that the open space may not be lost to the locality. The hospital's point of view is rather a hard one for, as Mr. Roberts has explained, the site in question is part of the hospital site, and was not intended as an open space—in fact, the governors have been paying undeveloped land duty on it. When the valuation of the site was recently laid before the London County Council, the committee offered to forego their right to build on the land if the Council would relieve them, as trustees of the hospital, of the monetary responsibility. This the Council was not prepared to do. Meanwhile the proposed hostel is important, not merely for the sake of the school, but indirectly for the nation, since the scarcity of medical men imposes a responsibility on the medical schools to make medical education and school life as attractive as possible. Every inch of space at St. Thomas's is now required, especially since the War Office has asked the governors, without limiting the accommodation for civilians, to provide 300 extra beds for military purposes. The real question to be answered is whether any other site is available, and if the hospital itself has no alternative position, and the Lambeth Council are sincere in their desire to retain the present open space, it should not be impossible, with mutual co-operation, to make an arrangement satisfactory to both parties. But time is short, and if a compromise is to be effected the Lambeth Council must be prepared to make the way smooth and the path easy.

### THE THEFT OF X-RAY APPARATUS.

In the case of the three men who, as reported in our columns, were lately charged respectively with stealing and receiving *x*-ray apparatus from different hospitals, judgment was delivered last week. Counsel for the prosecution pointed out that, as a man named De Redon had pleaded "Guilty" to stealing the apparatus, the jury would be concerned chiefly with the receiving of the goods by the two other defendants. The latter, he added, had the goods brought to them and purchased them for considerably less than cost price. The prisoners stated that the prices paid were fair prices for second-hand goods, and evidence was given in support of their contention by various auctioneers. The two recipients were found "Not guilty" and acquitted. On De Redon Judge Rentoul passed sentence of three months in the second division. One or two interesting minor points about the case may also be recorded. De Redon, in giving evidence for the prosecution, described himself as a traveller in electro-medical stores, in which capacity he had visited nearly every hospital in Great Britain. The man who was charged with receiving the goods carried on business as a dealer in electrical and scientific instruments, and in giving evidence on his own behalf stated that he had bought hundreds of *x*-ray tubes at different auction-rooms, where he had paid for them at the rate of three or four shillings each. The articles stolen included an *x*-ray tube from the Royal Free Hospital, an *x*-ray screen from Guy's, an electric battery from the London Homœopathic, besides the milliampere-meter mentioned previously.

### BRISTOL ROYAL INFIRMARY AS A BASE HOSPITAL.

Peculiar interest attached to the recent meeting of governors of the Bristol Royal Infirmary, since it was the first annual meeting to be held since the institution, under the scheme arranged in 1910, had been used as a Southern General Base Hospital. The still-remaining old institution has been preserved for the use of civil patients, over 4,000 of whom have received treatment; while in the new building, with its elaborate theatre units, which were fully described in The Hospital at the date of the opening, some 3,000 military patients have been received. The consequence was that Sir George White, the president, had most interesting material for his address, in which he pointed out that accommodation and cooking were provided for over 750 people a day, and that the infirmary had had as many as 500 in-patients at one time. The report stated that, in addition to the treatment of military out-patients, accommodation had been found for many wives and families of soldiers coming to England from India and the Colonies whose husbands had joined the Expeditionary Forces. The financial situation of the institution is also remarkable. The managers realised nearly all their investments in order to pay for the new building, and interest on investments only amounts to-day to £948. Sir George White's financial policy was to make good the loss of income from invested funds by a large roll of annual subscribers. Ten years ago there were about 1,400 annual subscribers; to-day there are 4,000, and the income from this source last year reached £7,775, an increase of £500 over the total raised in 1913. To complete this policy successfully £10,000 a year is required, and 2,000 more annual subscribers are urgently needed, since, as Sir George White pointed out, the hospital's capital indebtedness and revenue deficit amount in round figures to £30,000. All this becomes important in the light of the infirmary's extensive work as a base hospital, since the number of civil in-patients received during 1914 was 4,678, a decrease of 716; but exactly to what extent the number will be reduced remains to be seen in next year's report, when the war will have, as seems probable, lasted for twelve months. The total daily average number of occupied beds has risen from 270 in 1913 to approximately 450 for patients alone. The increase in the work is, therefore, as obvious as it is remarkable.

### NEW PRESIDENT OF THE ROYAL COLLEGE OF PHYSICIANS.

Sir Thomas Barlow's successor as President of the Royal College of Physicians is Dr. Frederick Taylor, M.D., F.R.C.P., the consulting physician to Guy's Hospital, at which he became a student after being educated at Epsom College. Born in 1847, the son of a medical man, Dr. Taylor graduated at London University, to which he was examiner in medicine from 1897-1906, and has been physician to the Seamen's Hospital, Greenwich, since 1896. He is also President of the Royal Society of Medicine, and his "Practice of Medicine" only last year ran into its tenth edition. Dr. Taylor has represented the University of London on the General Medical Council, and as a member of the Senate has done valuable work.

### THE FOURTH SERBIAN RELIEF UNIT.

The British Farmers' Hospital—that is to say, the fourth Serbian Relief Fund Unit—left London last week for Skoplje, Serbia, specially equipped to deal with cases of typhus, and under the charge of Mr. L. M. Wynch, C.I.E., the administrator. The equipment comprises a tent hospital for 300 beds, together with a complement of drugs and serums. Baths and an icing-plant were also taken. The staff includes four medical men, a secretary, and a dispenser, a matron, eighteen nursing sisters, thirteen women orderlies, and four male orderlies. An Admiralty transport is conveying the unit to Salonika, and it is interesting to note that a section of the third Serbian Relief Fund Unit—Mrs. Stobart's Hospital—consisting of a *personnel* of nineteen similarly bound, sailed in the same vessel. The rest of the third unit are travelling by the overland route. It is reported that Skoplje is full of typhus cases, and it is to be feared that the full extent of Serbia's needs is not yet appreciated in this country. The British Farmers' Red Cross Fund is still feeling the need for subscriptions, which can be sent to the secretary of the fund, Tower Bridge Flour Mills, Bermondsey. The accounts which have already come through indicate that Serbia is the one theatre of war in which the treatment of wounds, and still more of disease, has for various reasons lagged behind the modern measure of achievement, and the units which are now going out will probably have the most stringent experiences, as being the most needed of any.

### QUEEN ALEXANDRA AND THE ST. KATHARINE'S HOSPITAL SITE.

We have already recorded the fact that the Stepney Borough Council has petitioned Queen Alexandra, as patron of St. Katharine's Hospital, for a reconsideration of the decision of the Chapter to establish a college in Poplar, with a view to its establishment in Stepney. The Chapter Clerk has now informed the Council that by command of Queen Alexandra the petition was laid before the Chapter at its last meeting. "I am instructed to say in reply," he adds, "that the present site and buildings are entirely experimental. When funds permit, it is hoped that a permanent college may be erected, but the site for such permanent buildings has not yet even been discussed. When the time comes the Chapter will advise her Majesty to give any suggestions from the Borough of Stepney a full and careful hearing."

### THE RETURN TO WORK OF DISCHARGED CONSUMPTIVES.

Notice in a place by itself may be given to a list of suitable employments for patients suffering from tuberculosis, whose disease has been arrested or cured, which is contained in Dr. Harold Vallow's book on after-care, entitled "The Inevitable Complement." His experience as chief clinical tuberculosis officer for Bradford must be valuable, and the recommendations cited include domestic service for women where there are no children, and shop work for men. Nowadays, when there is such a reaction against domestic service, it is good to learn that the number of discharged patients whom Dr. Vallow has observed in this occupation has almost invariably justified the choice of it. Indeed it is obvious that the real objection to domestic service is not in the life itself, for those who show ability for it, but in the difficulty of guaranteeing a standard of comfort, health, good treatment, and prospects. Where the home is a good home in the medical and general sense, it is clear that, under the proviso of there being no children, home care, good food, not too tiring work, and a reasonable amount of fresh air should all await the girl who seeks employment on her discharge from a sanatorium. Domestic service may be the healthiest and most comfortable of lives for young women, in spite of its dulness and the fact that its work cannot be squeezed into definite hours like that of a factory; but it may be the reverse of all these things, and that it has been has no doubt produced the reaction against it. Shop work on the face of it has less to recommend it apart from the big advantage which it shares with all factories of a minimum standard being guaranteed. The counter and the office must always present difficulties to those who have suffered, or show signs of suffering, from tuberculosis; but that health can be preserved in these occupations is a tribute to the need for a still better organised system of after-care.

### THE EVOLUTION OF HOSPITAL ARCHITECTURE.

Before the Royal Institute of British Architects a paper was read last week by Mr. W. A. Pite on King's College Hospital at Denmark Hill. Hospital men have already taken so much interest in the new institution that it was hardly to be expected that even Mr. Pite, the architect, could have very many new features to point out. Still, as he remarked, the decision to remove from Lincoln's Inn Fields instead of remodelling the old buildings furnished the first example in the twentieth century of an important general hospital starting afresh on a different and distant site with a clean slate. Modern methods of cheap and quick transit had made it possible to move the hospital from the centre to the outskirts of London without at the same time moving it beyond the reach of thousands of sick poor. Indeed, we may add that it has rather followed the population than moved away from it. Though the "gaunt pile of Thomas Bellamy," in Mr. Pite's words, was no more, the fine traditions which grouped themselves around such names as those of Lister, Todd, Ferguson, and Priestly had transferred themselves to the new buildings. When history determines the place which new King's College Hospital holds in the evolution of hospital architecture, it may be that it will be regarded rather as the culmination of a tendency towards elaboration and magnificence of architecture than as the first example of a new type. As an illustration, the open-air treatment for almost every type of disease is slowly commending itself, with a greatly simplified architectural design as the result, and now that the European war is forcing on us the necessity for an enormous growth of hospital accommodation, largely on simple emergency lines, the future may well be to simple and relatively inexpensive hospital buildings. The natural temptation to glorify an institution to which a medical school is attached may prove less irresistible in days to come.

### ANOMALOUS SALARIES IN THE POOR-LAW MEDICAL SERVICE.

At a recent meeting of the Shoreditch Guardians a protest was made against the inconsistency of the Local Government Board in dealing with the salaries of medical officers at Metropolitan infirmaries. One of the members said that the Local Government Board had refused to allow the Guardians to offer a salary of £280 per annum for the post of first assistant medical officer, but had very soon afterwards sanctioned this amount being paid in the case of Bethnal Green. The result was that their first assistant had applied for the post and had been appointed, and the Shoreditch Guardians had thus lost an excellent officer and were faced with the task of finding a successor. The Guardians decided to approach the Local Government Board again for sanction to pay their first assistant medical officer a salary of £280 per annum. Anomalies of this kind are very common in the Poor-Law Medical Service. Different scales of salaries, in some cases widely divergent, are found in the London infirmaries, and are a source of much discontent. A more uniform system both as regards staffing and payment is urgently required.

### AN OFFICER'S MEMORIAL.

Already the endowment of a hospital bed is seen to figure as a fitting memorial for one who has died in the service of his country during the present war, since we learn that the Royal Berkshire Hospital at Reading has received the sum of £1,000 from Mrs. Jessie Anne Bruce, of Arborfield Court, Berks, to endow a bed in memory of her grandson, Frederic de Vere Allfrey, Lieutenant 9th Lancers, who was killed in action on September 7, 1914, near Provins, France.

### MEDICAL PRESSURE AT CAMBERWELL INFIRMARY.

Dr. W. J. C. Keats, medical superintendent of Camberwell Infirmary, has addressed a letter to the Camberwell Board of Guardians in which he points out the severe strain upon him owing to the non-appointment of a permanent resident assistant medical officer. The heavy additional work was likely to result in a serious breakdown of health to him, and, in order to avoid this, the Board should take steps to appoint an assistant who could help him in times of heavy work. The Guardians were fully alive to the difficulties of the situation so far as they affected Dr. Keats and the infirmary. The dearth of suitable men to take up the duties at the present juncture has also to be considered. The question of salary was also another drawback, but this the Board dealt with by deciding to offer an increased salary of £500 a year, with the usual emoluments. The successful candidate is to give an undertaking that he will not offer his services to the war authorities, but will occupy the position until the termination of the war. Extreme trouble has been experienced by the Camberwell Board of Guardians, in common with other public authorities, to retain the services of the medical staff, and men have resigned within a month after their appointment. It remains to be seen whether the present offer will result in the Board obtaining a suitable doctor. One reason adduced for offering the enhanced salary was that they were likely to get the services of an experienced doctor, and not a young man, who, however willing he might be, lacked the essential experience for such an office.

### A STRIKE AT A HOSPITAL.

Any history of the hospital emergency work undertaken through the war in this country will have to note the share which industrial discontent has taken in this activity also, though in a much less degree than in the case of the manufacture of munitions and the work at the docks. The strike which began just before Easter in connection with the work of converting the Stationery Office into a temporary War Hospital provides a case in point. The dispute arose as to what grade of workman should be employed to fit the water supplies. Fitters were engaged by the contractors, whereupon the plumbers went on strike on the ground that this was their work. It seems that the officers of the Operative Plumbers' Association had tried, without success, to secure a settlement. After the Government Committee on Production had considered the matter, work was agreed to till the settlement was announced. When the men presented themselves they were refused access to the work, and the other plumbers employed struck in sympathy.

### SHORT STAFFS AND THE ANNUAL HOLIDAY.

The majority of hospital and institutional bodies have now under consideration the question of annual leave for their officers. This year, owing to the exceptional dearth of medical practitioners, dispensers, nurses, and trained officers generally, it will be a matter of great difficulty to arrange for temporary workers. Indeed, in some institutions, with already greatly depleted staffs consequent on the demands of attending to the sick and wounded of our Army and Navy, the question of whether principal officers shall have their usual annual leave will have to be considered. Although in normal times the officers mentioned benefit greatly by a respite from the exacting work of administration, yet it must now be remembered that conditions are not at present normal. Should it prove necessary for officers to have a shorter period of annual leave this year, in the interests of the institution and its patients, loyal workers, we are sure, will recognise that by falling in with the arrangement they will be contributing their quota to the nation's cause. We understand that in some large business concerns the arrangement has been made for employees entitled, say, to fourteen days' leave to receive this year eight days, their salary, however, being paid for the full fortnight. If hospital workers are willing to accept some such proposal, we feel sure that their committees will at least agree to pay their holiday salary just as if they had taken full time leave. What are our readers' opinions ?

### A PRESENTATION FOR WAR SERVICES.

At last week's meeting of the council of the Hampstead General and North-West London Hospital Mr. Conrad W. Thies was invited to attend in order to receive the thanks of the council for his kind voluntary help during the absence of the late secretary and pending the appointment of his successor. The council passed the following resolution: "That the best thanks of the council be and they are hereby accorded to Mr. Conrad W. Thies for the great assistance he has rendered to the hospital during the absence of the late Mr. Thomas, and pending the appointment of his successor. The council beg Mr. Thies' acceptance of a gold watch, with suitable inscription, as a slight token of their appreciation of his services during a time of difficulty." An engrossed copy was handed to Mr. Thies by Mr. Charles Walton Sawbridge, the chairman, who further emphasised the council's gratitude, and at the same time presented to him the gold watch mentioned in the resolution. The watch was engraved with a monogram on the outer case and inscribed: "Presented to Conrad W. Thies, Esq., by the Hampstead General and North-West London Hospital in appreciation of his honorary services in the year 1914 during the absence of their secretary, who was killed in the great war."

### A MENTAL HOSPITAL ATTENDANT CHARGED.

An attendant at Leavesden Mental Hospital, King's Langley, Herts, named John Hubbard, aged sixty-eight, was charged before the Lord Mayor at the Mansion House on Tuesday with attempted suicide. The evidence showed that he was found by a police constable lying on the pavement near Fleet Street, bleeding from cuts in the right temple and left wrist. The cuts, according to a doctor, were apparently self-inflicted. After the chief clerk had recalled that the prisoner had been before the Court on remand on February 10 (having on that occasion taken laudanum), when he was discharged, he was committed for trial at the Old Bailey. Our readers will remember that Leavesden is the home of the living-out system, which has been advocated by Dr. F. A. Elkins, the medical superintendent, as an invaluable corrective to the depressing effects of the "asylum atmosphere." An account of the system was given in The Hospital two or three years ago.

### THE PRINCESS CHRISTIAN HOSPITAL TRAIN.

This train, which was open to inspection at Paddington Station on Tuesday before being shipped to France, is really a complete hospital unit on wheels. The necessary funds have been raised on the initiative of Princess Christian, under whose auspices a similar train was sent to South Africa in 1899. The experience thus gained has been utilised by Sir John Furley and Mr. William J. Fieldhouse, and the train has been constructed by the Birmingham Railway Carriage Company from the latter's design. According to War Office rules, each train must accommodate 400 patients of the cot and sitting-up class, and the mechanical details have had to be approved by the French railway engineers. There are eight coaches for cot cases, and four more, which were not on view, each for fifty sitting-up cases. To comply with French conditions, an ordinary brake van has to be added to each end, thus making fourteen coaches of a total length of about 700 feet. After the first brake van, the first coach contains an office, provided with a bed, fittings, desk, safe, etc., for the quartermaster-sergeant, partitioned off from a ward which contains beds for thirty patients. Nos. 2, 3, 6, and 7 have each thirty-six beds, with lavatory in the centre, and lockers in the four corners. No. 4 has beds for twelve orderlies, with two lockers and lavatory; a kitchen fully equipped and provided with a tank for 60 gallons of hot and 90 of cold water, and beyond this is a capacious store room for linen. The first part of No. 5 carriage affords sleeping accommodation for the nurses, and there are two compartments for nurses' and doctors' dining rooms, and an office with sleeping accommodation for the principal medical officer. Next to this is the surgery, followed by a sleeping room for doctors. No. 8 contains beds for twelve orderlies, a kit store, and a second kitchen, chiefly intended for hot water and small cooking. Each hospital coach is provided with 50 gallons of cold water and an extra tank containing 6 gallons of drinking water. The train is lighted with gas and electricity, and as a precaution gimble candle-lamps are placed in each carriage. The equipment includes straw mattresses, bedding, linen, china, plate, glass, cutlery, hot-water trays, and invalid tables.

### WOMEN AND HOSPITALS AFTER THE WAR.

The effect of the war in opening up the professions of medicine and nursing to women in the sense of providing not merely greatly increased opportunities, but also a strong demand for their services, is a feature the importance of which is not generally grasped. While critics affirm that the opportunities will be temporary, they forget that this is not exactly true, since, although on the declaration of permanent peace there will be no more wounded soldiers, the new numbers of women doctors and of nurses will have shown their value so prominently that the demand for their services will, in all probability, be made in quarters hitherto deaf to their usefulness. Moreover, the nation as a whole before the war was badly understaffed in respect of both professions—how badly the excess of sickness claims over those estimated by the actuaries of the Approved Societies remain to show. Thus the importance of hospital work, so much impressed on the whole community through the war, will give an impetus to medical work when it is over, and this impetus will be strengthened by numbers of medical women and nurses who, having been attracted to an interesting and valuable profession will not be at all anxious to give up their work and return home, or to an unoccupied life. We may thus expect the existing gaps in our organisation of school clinics, day nurseries, schools for mothers, maternity centres, and so on, to absorb a considerable portion of the energy now focussed on emergency hospital work. Therefore, when we hear that a women's war hospital is reported to be in process of formation in the recently closed Endell Street buildings, and that the War Office is lending support to a scheme for providing a relief hospital at an old house in Upper Tulse Hill, Brixton, entirely under the management of women, we may be sure that the clock cannot be wholly set back after the war. At Brixton the women workers are making themselves responsible for all expenses above the War Office allowance of three shillings per day per head. It is understood that all work will be honorary, even housework. To realise the true significance of women's emergency hospital work it is necessary to look beyond the individual schemes to which we are becoming accustomed by their number.

### THE LATE LORD ROTHSCHILD.

A grave loss to the world of charity and voluntary effort has occurred with the death of Lord Rothschild, on March 31, at the age of seventy-four. Apart altogether from the innumerable gifts to charity for which he was annually responsible, his work for hospitals has been very prominently in the public mind of late on account of his position as chairman of the Council of the British Red Cross Society. The now famous appeal on its behalf, signed by him, Sir Frederick Treves, and Mr. E. A. Ridsdale, which appeared in the *Times* of August 31 last, was followed by the inauguration of the *Times* Fund for the Sick and Wounded, which amounted on the day of his death to over £1,126,000. As treasurer from the inception of King Edward's Hospital Fund for London, he performed invaluable services, and he was also a member of the General Council. The voluntary system had no more powerful friend and no more consistent supporter than he. He was President of the Royal Hospital for Diseases of the Chest, the Royal Dental Hospital, and the Hospital for Diseases of the Throat, to give but three instances of his active interest in institutional affairs; and to show how alive he was to new needs as they arose, it may be mentioned that shortly before his death he subscribed £100 to the recently formed War Nurses Relief Fund, now to be known as the Queen Alexandra Relief Fund for War Nurses. It is hardly too much to say that the place which the Jewish community holds in relation to charity was largely due to his leadership, which, not content with that support that his wealth enabled him to give, comprised also a large degree of personal service of which his work for the King's Fund is perhaps the chief example.

### THIS WEEK'S DRUG MARKET.

Business was, of course, at a standstill during the Easter holidays, but the recovery from the holiday feeling was rapid and the market is now active again. The upward tendency of prices continues and the scarcity of some drugs is becoming more accentuated. Most of the synthetic drugs are still tending upwards in price. As was anticipated, quotations for potassium, sodium, and ammonium bromides and hydrobromic acid have been advanced; a further advance seems more probable than any decline. There is little change in the position of cod-liver oil, and the future of this market is doubtful; at the moment there are no signs that any substantial fall in price is pending, but the advance seems to have been checked for the time being. Oil of cloves is dearer. There has been a further advance in the price of cocaine, which is now quoted at more than three times the pre-war figure. Chlorate of potash and permanganate of potash have again advanced in price. Oil of sandalwood is dearer. A rise in the quotations for bismuth preparations is not unlikely.

